# Overexpression of a Stress-Responsive NAC Transcription Factor Gene *ONAC022* Improves Drought and Salt Tolerance in Rice

**DOI:** 10.3389/fpls.2016.00004

**Published:** 2016-01-22

**Authors:** Yongbo Hong, Huijuan Zhang, Lei Huang, Dayong Li, Fengming Song

**Affiliations:** National Key Laboratory for Rice Biology, Institute of Biotechnology, Zhejiang UniversityHangzhou, China

**Keywords:** abscisic acid (ABA), drought tolerance, NAC transcription factor, *ONA022*, salt tolerance, rice (*Oryza sativa* L.)

## Abstract

The NAC transcription factors play critical roles in regulating stress responses in plants. However, the functions for many of the NAC family members in rice are yet to be identified. In the present study, a novel stress-responsive rice NAC gene, *ONAC022*, was identified. Expression of *ONAC022* was induced by drought, high salinity, and abscisic acid (ABA). The ONAC022 protein was found to bind specifically to a canonical NAC recognition *cis*-element sequence and showed transactivation activity at its C-terminus in yeast. The ONAC022 protein was localized to nucleus when transiently expressed in *Nicotiana benthamiana*. Three independent transgenic rice lines with overexpression of *ONAC022* were generated and used to explore the function of *ONAC022* in drought and salt stress tolerance. Under drought stress condition in greenhouse, soil-grown *ONAC022*-overexpressing (N22oe) transgenic rice plants showed an increased drought tolerance, leading to higher survival ratios and better growth than wild-type (WT) plants. When grown hydroponically in Hogland solution supplemented with 150 mM NaCl, the N22oe plants displayed an enhanced salt tolerance and accumulated less Na^+^ in roots and shoots as compared to WT plants. Under drought stress condition, the N22oe plants exhibited decreased rates of water loss and transpiration, reduced percentage of open stomata and increased contents of proline and soluble sugars. However, the N22oe lines showed increased sensitivity to exogenous ABA at seed germination and seedling growth stages but contained higher level of endogenous ABA. Expression of some ABA biosynthetic genes (*OsNCEDs* and *OsPSY*), signaling and regulatory genes (*OsPP2C02*, *OsPP2C49*, *OsPP2C68*, *OsbZIP23*, *OsAP37*, *OsDREB2a*, and *OsMYB2*), and late stress-responsive genes (*OsRAB21*, *OsLEA3*, and *OsP5CS1*) was upregulated in N22oe plants. Our data demonstrate that *ONAC022* functions as a stress-responsive NAC with transcriptional activator activity and plays a positive role in drought and salt stress tolerance through modulating an ABA-mediated pathway.

## Introduction

Plants have developed an array of sophisticated mechanisms at multiple levels to cope with unfavorable environmental stresses, such as drought and high salinity (Yamaguchi-Shinozaki and Shinozaki, 2006). Upon perception of external stress signals, a complicated signaling network is effectively and timely initiated, which ultimately reprograms the expression of a large set of stress-responsive genes (Zhu, 2002; Shinozaki and Yamaguchi-Shinozaki, 2007; Golldack et al., 2014; Nakashima et al., 2014). This expression reprograming of stress-responsive genes at genome-wide level often activates various cellular, physiological, biochemical, and metabolic processes, including stomatal closure, repression of cell growth and photosynthesis, activation of respiration, and accumulation of compatible osmolytes, such as proline and soluble sugars, which protect plants from damages and thus increase the chance of survival (Shinozaki and Yamaguchi-Shinozaki, 2007; Hu and Xiong, 2014).

Recent extensive genetic and molecular studies using knockout/knockdown mutants and overexpression transgenic lines in model plants as well as in crop plants have demonstrated that many transcription factors (TFs) belonging to the NAC, AP2/ERF, MYB, WRKY, bZIP, homeodomain, bHLH, NF-Y, and CAMTA families play important roles in plant responses to abiotic and biotic stresses (Ariel et al., 2007; Chen et al., 2012; Mizoi et al., 2012; Puranik et al., 2012; Rushton et al., 2012; Licausi et al., 2013; Nuruzzaman et al., 2013; Castilhos et al., 2014; Nakashima et al., 2014; Shao et al., 2015). The NAC (NAM, AFAT, and CUC) proteins are characterized with a conserved region, called NAC domain, at their N-terminals and a highly divergent C-terminus (Olsen et al., 2005). Genome-based bioinformatics analyses have showed that the NAC proteins constitute a large plant-specific family with more than 100 members (Ooka et al., 2003; Fang et al., 2008; Nuruzzaman et al., 2010; Le et al., 2011). A large number of the NAC TFs have been characterized for their roles in plant abiotic stress responses (Puranik et al., 2012; Nuruzzaman et al., 2013). Transcriptional profiling analysis revealed that at least 33 *Arabidopsis* NAC genes were responsible to abiotic stresses including high salinity (Jiang and Deyholos, 2006). Similarly, it was also found that a large portion of the rice NAC family exhibited overlapping expression patterns in rice under various abiotic and biotic stresses (Nuruzzaman et al., 2015; Sun et al., 2015). Six rice NAC genes have been shown to be involved in defense response against pathogen infection, e.g., *ONAC048* (*OsNAC6*), *ONAC048* (*OsNAC111*), *ONAC122*, and *ONAC131* in regulating defense response against *Magnaporthe oryzae* causing blast disease (Nakashima et al., 2007; Sun et al., 2013; Yokotani et al., 2014), *ONAC054* (*RIM1*) with negative effect on resistance to rice dwarf virus (Yoshii et al., 2009) and *ONAC068* (*OsNAC4*) as a positive regulator of hypersensitive cell death (Kaneda et al., 2009). The involvement of NAC TFs in rice abiotic stress response was extensively explored and seven NAC genes have been characterized to play important roles in abiotic stress tolerance in rice. For example, overexpression of *ONAC002* (*SANC1*/*OsNAC9*), *ONAC048* (*SNAC2*/*OsNAC6*), *ONAC009* (*OsNAC5*), *ONAC122* (*OsNAC10*), *ONAC045*, or *ONAC058* (*OsNAP*) improved significantly the drought and salinity tolerance in transgenic rice (Hu et al., 2006, 2008; Nakashima et al., 2007; Zheng et al., 2009; Jeong et al., 2010, 2013; Takasaki et al., 2010; Song et al., 2011; Redillas et al., 2012; Chen et al., 2014; Liang et al., 2014) and some of these transgenic rice lines showed increased drought tolerance under severe drought stress conditions without any adverse effect on yield or even with yield increase (Hu et al., 2006; Jeong et al., 2010, 2013; Redillas et al., 2012; Chen et al., 2014). Abscisic acid (ABA) is a well-known stress hormone that coordinates the complex networks of stress responses. ABA-mediated signaling pathway plays important roles in abiotic stress responses regulated by several NAC TFs in transgenic rice plants, as demonstrated by the hypersensitivity to ABA (Hu et al., 2006, 2008; Chen et al., 2014), upregulated expression of ABA biosynthesis-related genes (Jeong et al., 2010; Redillas et al., 2012), increased endogenous ABA level (Liang et al., 2014), and upregulated expression of a set of ABA-responsive stress-related genes (Nakashima et al., 2007; Hu et al., 2008; Jeong et al., 2010, 2013; Takasaki et al., 2010; Redillas et al., 2012; Chen et al., 2014). In addition, altered stomatal movement or root system regulated by overexpression of *ONAC002* (*SANC1*/*OsNAC9*), *ONAC009* (*OsNAC5*), or *ONAC122* (*OsNAC10*) was also found to be involved in the improved abiotic stress tolerance of the transgenic plants (Hu et al., 2006; Jeong et al., 2010, 2013; Takasaki et al., 2010; Redillas et al., 2012).

We are interested to characterize novel TF genes that regulate plant stress response and try to generate stress-resistant/tolerant crops through transgenic approach. In our previous study, we characterized a number of stress-responsive *ONAC* genes in rice response to biotic and abiotic stresses using publicly available microarray data (Sun et al., 2013, 2015) and noticed that one *ONAC* gene, *ONAC022* (LOC_Os03g04070), was significantly induced by several abiotic and biotic stress treatments. In the present study, we performed a detailed functional analysis by overexpression of *ONAC022* in transgenic rice to explore its roles in abiotic stress tolerance.

## Materials and Methods

### Plant Growth and Treatments

Rice (*Oryza sativa* L.) cultivar Yuanfengzao was used for analyses of gene expression in response to abiotic stress and ABA treatment whereas cultivar Xiushui 134 for generation of transgenic lines and phenotype analyses. Seedlings were grown in a soil mix (clay is mixed with soil at 3:1 ratio) in a growth room under daily cycle of 28°C 14 h light (>3000 lux)/22°C 10 h dark or in a greenhouse with natural sunlight. For hormone treatment, 2-week-old seedlings grown in a growth room were treated by spraying with 100 μM ABA or with equal volume of solution containing only 0.1% ethanol and 0.02% Tween-20 as controls. For drought stress treatment, seedlings were put on lab blenches without water supply or on water-saturated filter papers in Petri dishes as controls. For salt stress treatment, seedlings were irrigated with 200 mM NaCl solution. Leaf samples were collected and stored at –80°C until use.

### Cloning and Sequence Analysis of *ONAC022*

Based on the predicted sequence of *ONAC022* (LOC_Os03g04070) in Rice Genome Annotation database and a full-length cDNA (AK107090) in GenBank database, the coding sequence of *ONAC022* was amplified using a pair of gene-specific primers (Supplementary Table S1) and cloned into pMD19-T vector, yielding plasmid pMD19-ONAC022. After confirmation by sequencing, plasmid pMD19-ONAC022 was used as a template for all experiments. Multiple sequence alignment was performed using ClustalW program in the Lasergene software. Phylogenetic tree analysis with other rice NAC proteins retrieved from the Rice Genome Annotation database was performed using neighbor-joining method in MEGA5 software with 1000 replications. The promoter sequence of the *ONAC022* gene (1500 bp upstream from the transcription start site) was retrieved and searched for putative *cis*-elements at the PLACE database (http://www.dna.affrc.go.jp/PLACE/signalscan.html).

### Subcellular Localization Assay

The coding sequence of *ONAC022* was amplified using a pair of gene-specific primers (Supplementary Table S1) and inserted into vector pFGC-EGFP at *Bam*HI/*Xba*I sites, yielding plasmid pFGC-GFP-ONAC022. This plasmid and the pFGC-EGFP empty vector were transformed into *Agrobacterium tumefaciens* GV3101 and the agrobacteria were infiltrated separately into leaves of *Nicotiana benthamiana* plants expressing a red nuclear marker RFP–H2B protein (Chakrabarty et al., 2007) using 1-mL needless syringes. After agroinfiltration, the plants were grown in a growth room under 25°C for 48 h. GFP fluorescence signals were excited at 488 nm and detected under a Zeiss LSM 510 Meta confocal laser scanning microscope (Oberkochen, Germany) using a 500–530 nm emission filter.

### DNA Binding and Transactivation Activity Assays

For DNA binding activity assay, the coding sequence of *ONAC022* was amplified using a pair of gene-specific primers (Supplementary Table S1) and inserted into pGEX-6p-1 (GE Healthcare, Piscataway, NJ, USA) at *Bam*HI/*Eco*RI sites, yielding plasmid pGEX-6p1-ONAC022, which was then transformed into *Escherichia coli* strain BL21. GST-fused ONAC022 recombinant protein was purified using glutathione resin column (Genscript, Shanghai, China) according to the manufacturer’s protocol. A 28 bp *cis*-element fragment wNACRS (ATCGCATGTGGAGCACGGAGCACGTTTT, the core sequences underlined; Xie et al., 2000; Trans et al., 2004) and a mutated fragment mNACRS (ATCGAAAAAAGAGAAAAGAGAAAATTTT, the mutated nucleotides underlined) were labeled with biotin. Electrophoretic mobility shift assay (EMSA) was performed using a chemiluminescent EMSA Kit (Beyotime Biotechnology, Haimen, China). Binding reactions were conducted in a total of 10 μL volume containing 5x EMSA buffer, 2 μg recombinant ONAC022 protein or GST protein (as a negative control) and 1 μL biotin-labeled wNACRS or mNACRS probe. For the competitive reactions, excess unlabeled wNACRS or mNACRS probe (in excess of 200 times) was added and incubated for 20 min before addition of the labeled wNACRS probe. The reaction mixtures were separated on 8% native PAGE and transferred onto nitrocellulose membranes. Signals from the probes were detected according to the manufacturer’s protocol. For transactivation activity assay, the coding sequence of *ONAC022* was amplified using a pair of gene-specific primers (Supplementary Table S1) and fused in-frame to yeast GAL4 DNA binding domain in vector pBD-GAL4Cam, yielding plasmid pBD-ONAC022. Plasmid pBD-ONAC022 and pBD empty vector were transformed into yeast strain AH109. The transformed yeasts were plated on SD/Trp^-^ or SD/Trp^-^ His^-^ medium and incubated for 3 days at 30°C, followed by addition of x-α-gal. Transactivation activity of the fused proteins was evaluated according to the growth and production of blue pigments after addition of x-α-gal on SD/Trp^-^ His^-^ medium.

### Generation and Characterization of N22oe Lines

The coding sequence of *ONAC022* was amplified using ONAC022-F-OE and ONAC022-R-OE (Supplementary Table S1) and inserted into binary vector pCoUm (Zhang et al., 2008) under the control of a maize *ubiquitin* promoter, yielding plasmid pCoUm-Ubi::ONAC022. The resulting construct was introduced into rice calli of cultivar Xiushui134 through *Agrobacterium*-mediated transformation. T2 generation of the obtained N22oe lines was screened by planting seeds on 1/2 MS medium supplemented with 50 μg/L hygromycin (Hgr) and lines showing 3:1 (Hgr-resistant:Hgr-susceptible) segregation were selected as putative transgenic lines with single-copy of the transgene. Seeds from individual lines of T3 generation were observed on selective medium and those that showed 100% resistance to Hgr were selected as homozygous lines. Homozygous lines with single-copy of transgene were used for all experiments. To further characterize these single-copy transgenic lines, genomic DNA was extracted with two volumes of 2x CTAB extraction buffer (2% CTAB, 10 mM Tris-HCl pH8.0, 20 mM EDTA pH8.0, 1.4 M NaCl, 2% 2-ME). Fifty micrograms of genomic DNA were digested completely with *Eco*RI, separated by electrophoresis on a 0.8% agarose gel, and transferred by capillary action overnight onto a Hybond-N^+^ nylon membrane (Amersham Biosciences, Little Chalfont, UK) using 20X SSC solution. A 589 bp fragment of the *Hpt*II gene was amplified using a pair of primers HptII-Probe-F and HptII-Probe-R (Supplementary Table S1) and labeled with DIG by the random priming method using a DIG High Prime DNA Labeling and Detection kit (Roche Diagnostics, Shanghai, China). Prehybridization, hybridization, and detection were performed according to the manufacturer’s recommendations.

### Phenotype Analyses for Drought and Salt Tolerance and ABA sensitivity

For drought tolerance assay, ten 4-week-old N22oe and wild-type (WT) seedlings were grown in the same pot with three replicates and were subjected to drought stress treatment by withholding watering for 15 days, followed by recovery with normal water supply for another 7 days. The survival ratio and fresh weight of the plants were calculated at 12 days after re-watering. Plants that were green and healthy young leaves after re-watering were regarded as survivals. Survival ratio was calculated as the ratio of number of survived plants over the total number of treated plants. For salt tolerance assay, 100 seeds were germinated on 1/2 MS medium supplemented with or without 150 mM NaCl under 28°C/25°C (day/night) with a 12 h photoperiod. At 6 days after germination, shoot height and fresh weight of at least 30 seedlings of each line were measured. For ABA sensitivity assay, 60 seeds were germinated on 1/2 MS medium containing different concentrations of ABA under 28°C/25°C (day/night) with a 12 h photoperiod. At 6 days after germination, the germination rates and shoot and root lengths for each line were recorded.

### Physiological and Biochemical Measurements

Content of free proline in leaves of rice plants was determined as previously described (Bates et al., 1973). Briefly, leaves were harvested, weighed, and extracted in 3% sulfosalicylic acid. An aliquot of each extract (2 mL) was incubated with 2 mL of ninhydrin reagent [2.5% (w/v) ninhydrin, 60% (v/v) glacial acetic acid, and 40% 6 M phosphoric acid] and 2 mL of glacial acetic acid at 100°C for 40 min, and the reaction was terminated on ice bath. Four milliliters of toluene solution were added to the reactions, followed by vortex and incubation at 23°C for 30 min, followed by measurement of the absorbance at 520 nm. Content of total soluble sugars in leaves of rice plants was measured as previously described (Bailey, 1958). Leaf samples were weighed and then extracted by 80% ethanol at 80°C for 30 min with occasional agitation. The supernatant was filtered and brought to a final volume of 10 mL with 80% ethanol. One milliliter of the extract was incubated with 5 mL anthrone reagent at 95°C for 15 min, and then the reaction was terminated on ice, followed by measurement of the absorbance at 620 nm. Relative water content (RWC) in leaves of rice plants was measured as previously described (Schonfeld et al., 1988). Fully expanded leaves from three- to four-leaf stage soil-grown plants were detached to measure the leaf fresh weight (*W*_F_), turgid leaf weight (*W*_T_), and dry weights (*W*_D_), and *RWC* were calculated from the equation

$$RWC\,\;\;(\%)=(W_{F}-W_{D})/(W_{T}-W_{D})\times 100\%.$$

Transpiration rate in leaves was measured using LI-6400 Photosynthesis System (LI-COR, Lincoln, NE, USA). Five flag leaves of 3-month-old plants grown in greenhouse were enclosed in a leaf chamber with a built-in red and blue light source at PAR of 800 μmol⋅m^-2^⋅s^-1^ in the gas exchange analyzer. The chamber CO_2_ concentration was controlled at 390 ppm using an automatic CO_2_ controller in the LI-6400 system. Measurements were performed at 10:00 AM, 12:00 PM, 14:00 PM, and 16:00 PM in a clear day for each leaf and at least eight measurements for each plant at each time point were collected to calculate the transpiration rate. Content of Na^+^ in root and shoot tissues was analyzed according to a previously described protocol (Jabeen et al., 2014). Briefly, shoots and roots were harvested from at least ten 4-week-old N22oe and WT seedlings grown hydroponically in modified Hogland solution supplemented with or without 150 mM NaCl. Samples were washed three times with deionized water, grinded to fine powder with liquid nitrogen and then dried at 70°C for 3 days. After weighting, the dried samples were digested with 10 mL nitric acid until clarification and the Na^+^ content was measured using a flame atomic absorption spectrometry (AA6300, Shimadzu, Japan). Stomatal closure was examined according to the method described previously (You et al., 2013). Briefly, fully expanded leaves from 8-week-old plants grown under normal or drought stress condition were detached and fixed in 2.5% glutaraldehyde solution. Stomatal status was monitored by scanning electron microscopy (SU8010, Hitachi, Japan).

### Quantification of Endogenous ABA Content

Leaf tissues (300 mg) were ground in liquid nitrogen. The powder was extracted with 2 mL 80% methanol and kept overnight at –20°C, followed by centrifugation at 4°C for 10 min at 12,000 × *g*. The supernatant was collected, dried under nitrogen gas and dissolved in 0.5 mL 2% ammonia solution. Crude extracts were further purified by Oasis MAX SPE columns (Waters Corp., Milford, MA, USA) that were sequentially preconditioned with 2 mL methanol and 2 mL 2% ammonia solution. After the supernatant was loaded, the columns were sequentially washed with ammonia solution (2%) and 2 mL methanol. ABA was eluted with 4 mL methanol containing 1% formic acid. The eluent was dried under nitrogen gas and dissolved in 0.5 mL 80% methanol. Quantification of ABA was performed by a HPLC-Triple quadrupole liquid chromatography-mass spectrometry system (Model 1290/6460, Agilent Technologies, Santa Clara, CA, USA) with stable isotope-labeled ABA as a standard according to a previously described method (Fu et al., 2012).

### qRT-PCR for Gene Expression Analysis

Total RNA was extracted from frozen leaf samples using TRIzol reagent (Invitrogen, Shanghai, China) and then treated with RNase-free DNase (TaKaRa, Dalian, China). First-strand cDNA was synthesized from 1 μg of total RNA using AMV reverse transcriptase (TaKaRa, Dalian, China) according to the manufacturer’s recommendation. qRT-PCR was performed on a CFX96 real-time PCR system (Bio-Rad, Hercules, CA, USA) using Fast Essential DNA Green Master kit (Roche Diagnostics, Shanghai, China). Each qRT-PCR reaction contained 12.5 μL 2x Fast essential buffer (Roche Diagnostics, Shanghai, China), 1 μg cDNA and 10 μmoL of each of gene-specific primers in a final volume of 25 μL. A rice *Actin* gene was used as an internal control to normalize the data and relative expression levels of genes of interest were calculated using the 2^-ΔΔCT^ method. Gene-specific primers used in qRT-PCR are listed in Supplementary Table S1.

### Microarray Analyses of Gene Expression Profiling

Leaf samples were collected from 3-week-old N22oe and WT plants and microarray analyses were performed using Affymetrix Rice Genome Array by standard protocol. All procedures for total RNA extraction, probe preparation, hybridization, scanning, data collection, and bioinformatics analyses were carried out at Beijing Capitalbio Biotechnology Company Ltd (Beijing, China). Genes with a twofold change in the expression level between N22oe and WT plants were defined as differentially expressed genes.

### Statistical Analysis

All experiments were repeated independently for at least three times and data were subjected to statistical analysis using the Student’s *t*-test at *p* = 0.05 level.

## Results

### *ONAC022* is a Stress-Responsive NAC Gene in Rice

**FIGURE 1:**
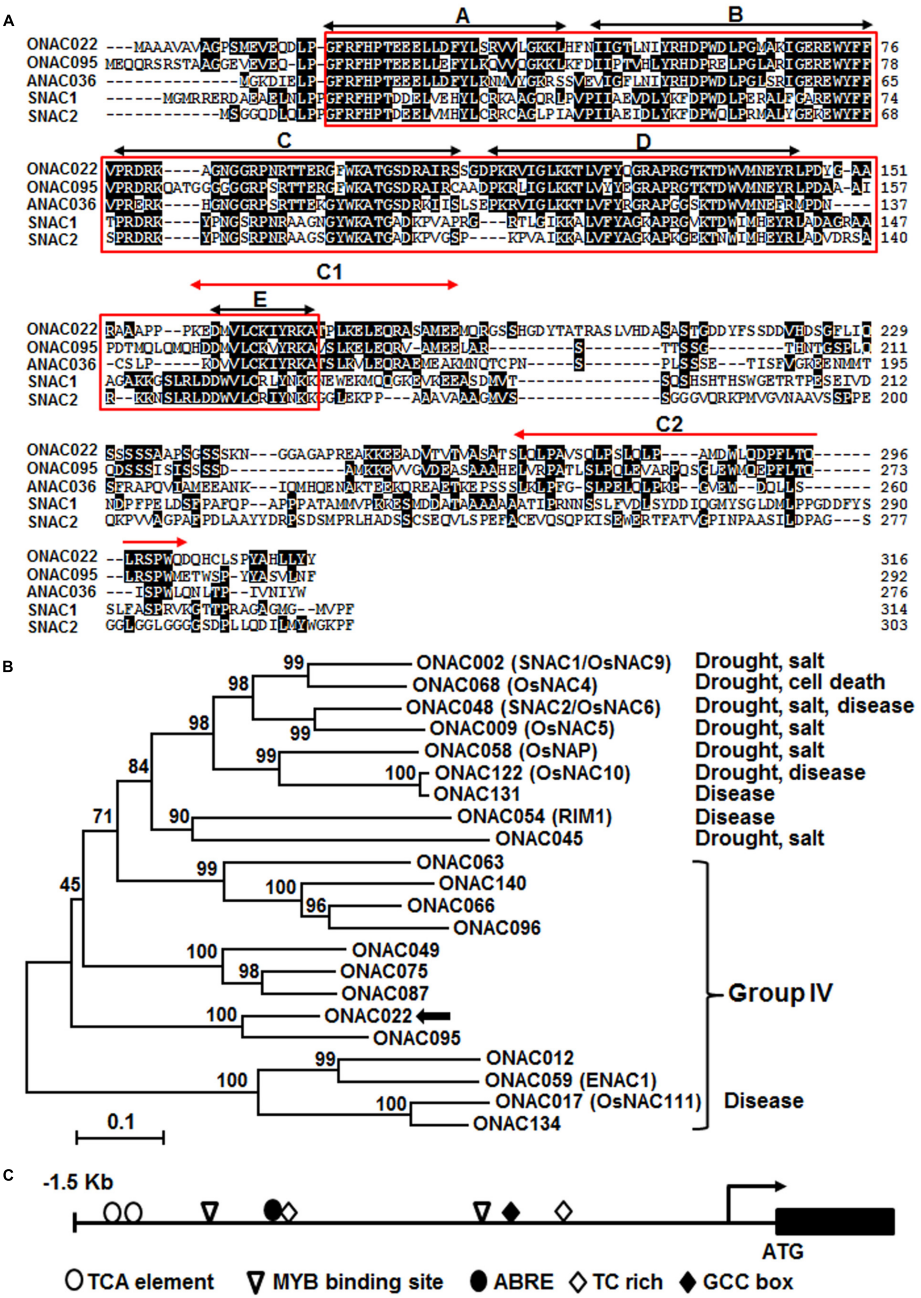
**Characterization of rice ONAC022 protein and gene. (A)** Alignment of ONAC022 with rice ONAC095, SNAC1 (OsNAC9), and SNAC2 (OsNAC6) and *Arabidopsis* ANAC036. Identical amino acids are shaded in black and the conserved NAC domain is boxed. Black arrowed lines indicate the locations of the five highly conserved subdomains A–E, while red arrowed lines show the locations of the newly identified C1 and C2 domains. **(B)** Phylogenetic tree analysis of ONAC022 with other known stress-responsive rice NAC proteins. Sequence alignment was performed using Clustal X1.81 program and phylogenic tree was created and visualized using MEGA 5.05. Protein sequences used for alignment are as follow: ONAC012 (Os05g37080), ONAC022 (Os03g04070), ONAC017 (Os11g05614), ONAC049 (Os08g02160), ONAC059/ENAC1 (Os01g64310), ONAC063 (Os08g33910), ONAC066 (Os03g56580), ONAC075 (Os01g66490), ONAC087 (Os05g34600), ONAC095 (Os06g51070), ONAC096 (Os07g04560), ONAC134 (Os12g05990), ONAC140 (Os12g43530), ONAC002/SNAC1/OsNAC9 (Os03g60080), ONAC048/SNAC2/OsNAC6 (Os01g66120), ONAC058/OsNAP (Os03g21060), ONAC122/OsNAC10 (Os11g03300), ONAC131 (Os12g03040), ONAC068/OsNAC4 (Os01g60020), ONAC009/OsNAC5 (Os11g08210), ONAC054/RIM1 (Os03g02800), ONAC045 (Os11g03370). Reported names and functions in stress response of the known stress-responsive NAC genes were given in parentheses in the tree and listed at right of the tree, respectively. **(C)** Distribution of major stress-related *cis*-elements in the promoter region of the *ONAC022* gene.

**FIGURE 2:**
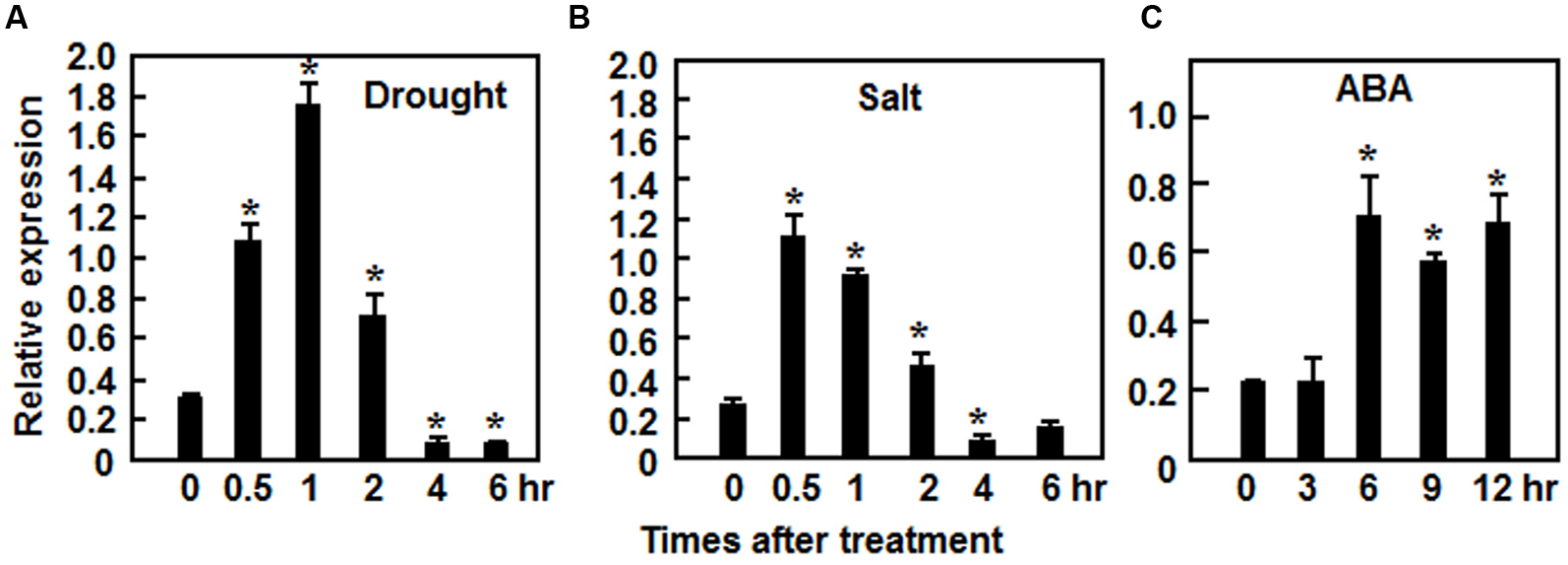
**Induction of *ONAC022* expression by drought, salt, and abscisic acid (ABA)**. Two-week-old seedlings were exposed to drought **(A)**, 150 mM NaCl **(B)**, or treated by foliar spraying with 100 μM ABA **(C)** and leaf samples were collected at indicated time points for analyses of gene expression by qRT-PCR. Relative expression levels as compared to those of the actin gene at each time point are presented as the means ± SD from three independent experiments. ^∗^Above the columns indicate significant differences at *p* ≤ 0.05 level with corresponding controls.

The *ONAC022* gene encodes a 316 aa protein with a typical NAC domain, which can be divided into five subdomains, namely A, B, C, D, and E (Ooka et al., 2003) (**Figure 1A**). In addition, the C-terminal region of the ONAC022 protein also contains newly identified unique conserved C1 and C2 domains that are absent in other NAC proteins (Kato et al., 2010) (**Figure 1A**). Phylogenetic analysis suggests that ONAC022 belongs to Group B (Nuruzzaman et al., 2010) or Phylogeny Group IV (Fang et al., 2008), which contains 14 members (**Figure 1B**). Phylogenetically, ONAC022 is closely related to rice ONAC095 and *Arabidopsis* ANAC036, showing 62 and 52% of identity at amino acid level, respectively, but is less related to other known stress-responsive NAC proteins in rice (**Figure 1B**). Bioinformatics analysis indicates that the promoter region (1.5 Kb upstream of the start codon) of the *ONAC022* gene contains some stress response-related *cis*-elements, including one ABRE element, two MYB binding sites, two TCA elements, one TC-rich element and one GCC box (**Figure 1C**). To explore the possible involvement of *ONAC022* in abiotic stress response, we analyzed the expression patterns of *ONAC022* in rice plants after treatment with abiotic stresses and ABA. qRT-PCR analyses revealed that the expression of *ONAC022* was significantly and rapidly induced within 2 h after salt and drought treatment, leading to fourfold to sixfold of increases (**Figures 2A,B**). Treatment of rice plants with ABA induced the expression of *ONAC022*, showing threefold to fourfold of increase during 6–12 h after treatment (**Figure 2C**). These data indicate that *ONAC022* is a stress-responsive NAC gene in rice.

### ONAC022 is a Nucleus-Localized Transcriptional Activator

**FIGURE 3:**
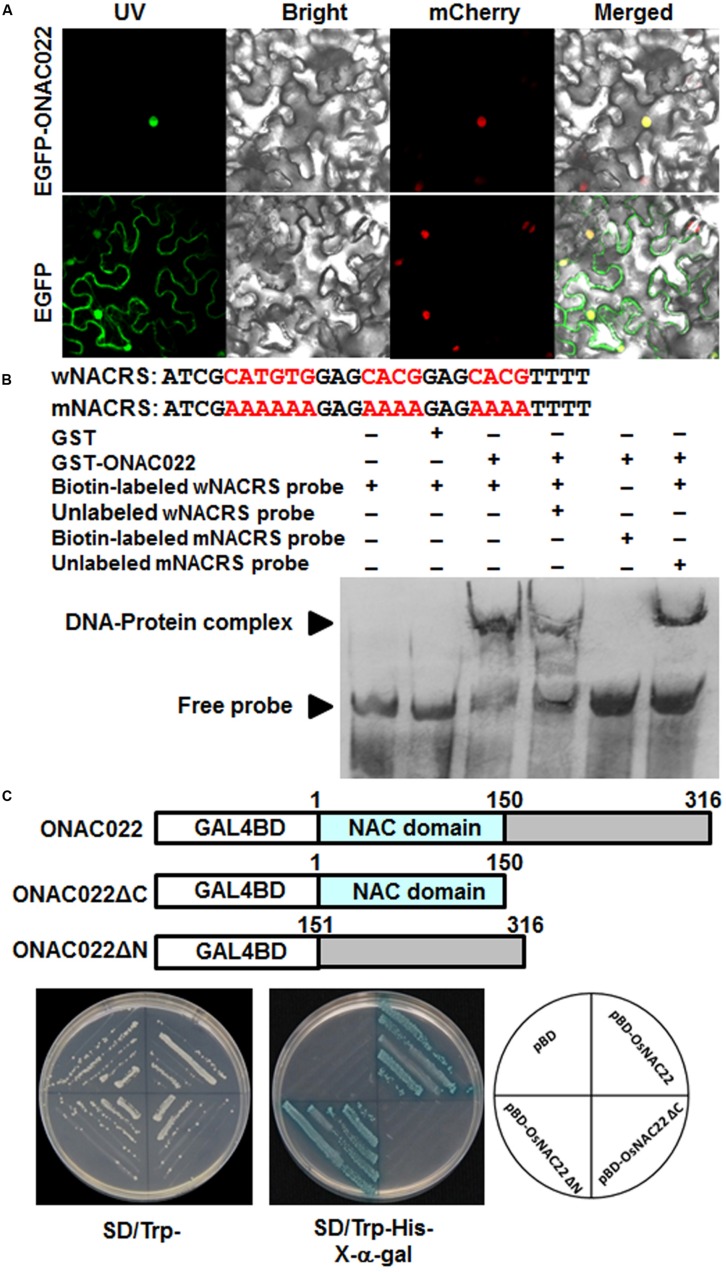
**Subcellular localization and transactivation activity of the ONAC022 protein. (A)** ONAC022 is localized in nucleus. *Agrobacteria* carrying pFGC-Egfp-ONAC022 or pFGC-Egfp empty vector were infiltrated into leaves of *Nicotiana benthamiana* plants expressing a red nucleus marker protein RFP–H2B and leaf samples were collected at 24 h after infiltration for observation under a confocal laser scanning microscope. Images were taken in dark field for green fluorescence (*left*) and red fluorescence (*middle right*), white field for cell morphology (*middle left*) and in combination (*right*), respectively. **(B)** DNA binding activity of ONAC022. Wild type version of the *cis*-element sequence (wNACRS) and a mutated version (mNACRS) were used. Electrophoretic mobility shift assays were performed using the recombinant GST-fused ONAC022 protein. Biotin-labeled wNACRS and mNACRS probe and biotin-labeled wNACRS probe in combination with unlabeled wNACRS or mNACRS probe were incubated with GST-fused ONAC022 protein or a purified GST preparation as a negative control. Specific DNA-protein complexes and free probes are indicated by the arrowheads on left. **(C)** ONAC022 has transactivation activity. Yeast cells carrying pBD-ONAC022, pBD-ONAC022ΔC, pBD-ONAC022ΔN or pBD empty vector (as a negative control) were streaked on SD/Trp^-^ plates (*left*) or SD/Trp^-^His^-^ plates supplemented with x-α-gal (*right*) for 3 days at 28°C.

To examine the subcellular localization of ONAC022, agrobacteria carrying pFGC-EGFP:ONAC022 and pFGC-EGFP (as a negative control) were infiltrated into leaves of 4-week-old *Nicotiana benthamiana* plants that expressed a red nuclear marker RFP–H2B protein (Chakrabarty et al., 2007). Confocal micrographs showed that the GFP:ONAC022 fusion protein was solely and clearly localized to the nucleus, co-localized with the known nucleus marker RFP–H2B protein (**Figure 3A**), whereas the GFP alone distributed ubiquitously throughout the cell without specific compartmental localization (**Figure 3A**). These data demonstrate that the ONAC022 protein is localized to nucleus of the cells.

To examine whether the ONAC022 protein had DNA binding activity, GST-ONAC022 fusion protein was purified to homogeneity in native PAGE. EMSA results revealed that the GST-ONAC022 protein bound to the biotin-labeled wNACRS fragment, which contained the CATGTG and CACG core motifs (Xie et al., 2000; Trans et al., 2004), forming a specific DNA-protein complex, but did not bind to the biotin-labeled mNACRS fragment (**Figure 3B**), in which the CATGTG and CACG motifs were replaced with the sequences AAAAAA and AAAA, respectively. In the competition binding assay, binding of GST-ONAC022 to the labeled wNACRS fragment was attenuated in the presence of excess unlabeled wNACRS fragment in the reaction but was not affected by unlabeled mNACRS fragment (**Figure 3B**). The purified GST protein did not bind to the wNACRS fragment (**Figure 3B**). These results indicate that the ONAC022 protein could specifically bind to the *cis*-element NACRS of the NAC proteins.

To determine whether ONAC022 had transcriptional activator activity, the full ONAC022 protein, an N-terminal fragment ONAC022ΔC (lacking 151-316 aa at C-terminal) and a C-terminal fragment ONAC022ΔN (lacking 1-150 aa at N-terminal) were each fused to the GAL4 DNA-binding domain of the pBD vector (**Figure 3C**). All yeast transformants grew well in SD/Trp^-^ medium (**Figure 3C**). On SD/Trp^-^His^-^ medium, only transformants carrying pBD-ONAC022 or pBD-ONAC022ΔN grew and showed β-galactosidase activity, whereas transformants carrying pBD-ONAC022ΔC and pBD empty vector did not (**Figure 3C**). These results indicate that the ONAC022 protein is a transcriptional activator and that the C-terminal region of ONAC022 is required for its transactivation activity.

### Generation of the *ONAC022*-Overexpressing (N22oe) Transgenic Rice

**FIGURE 4:**
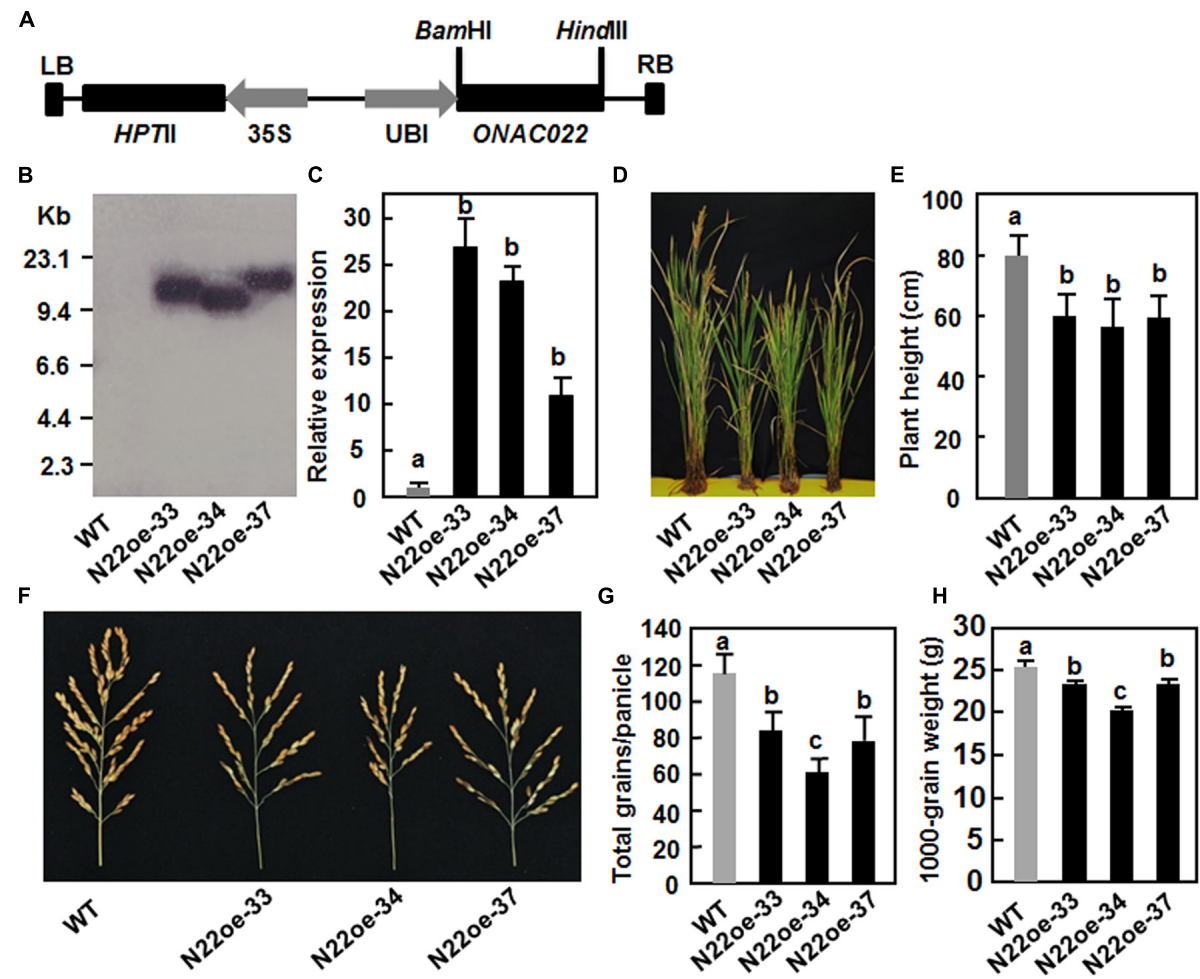
**Characterization of the *ONAC022*-overexpressing transgenic rice lines and their agronomic traits. (A)** Schematic diagram of the overexpression construct used for rice transformation. HptII, hygromycin (Hgr) phosphotransferase II; LB, left border; RB, right border; Ubi, maize ubiquitin promoter; 35S, CaMV 35S promoter. **(B)** Southern blot analysis of copy number of the transgene in T4 generation of the N22oe lines. Genomic DNA (∼50 μg) extracted from the N22oe and wild-type (WT) plants was digested with *Eco*RI and probed with a DIG-labeled fragment of the *Hpt*II gene. **(C)** Expression levels of the *ONAC022* gene in the N22oe lines. **(D)** Growth phenotypes of the N22oe plants at heading stage under normal watered condition in greenhouse. **(E)** Reduced plant height of the N22oe plants grown under normal watered condition in greenhouse. **(F)** Comparison of the panicles between the N22oe and WT plants grown under normal watered condition in greenhouse. **(G)** Numbers of grains per panicle between the N22oe and WT plants grown under normal watered condition in greenhouse. **(H)** Weights of 1000-grain from the N22oe and WT plants grown under normal watered condition in greenhouse. Data presented **(C,E,G,H)** are the mean ± SD from three independent experiments and different letters above the columns indicate significant differences at *p* ≤ 0.05 level with corresponding WT.

The *ONAC022* gene was transformed into rice cv. Xiushui134 under the control of the maize ubiquitin promoter (**Figure 4A**). A total of 23 independent transgenic lines were generated. After screening by Hgr resistance phenotype on 1/2 MS medium, three independent transgenic lines (N22oe-33, 34, and 37) were selected as candidates of single copy lines and were confirmed by Southern blotting with a fragment of the *Hgr* gene as a probe. Because there is no *Eco*RI sites in the region between LB and RB in the pCoUm vector, a single band of the *Eco*RI-digested genomic DNA from each of these three lines was detected (**Figure 4B**), indicating that the transgenic lines N22oe-33, 34, and 37 contained a single copy of the transgene. qRT-PCR analysis showed that the *ONAC022* gene was expressed normally in the transgenic lines (T3 generation) and the expression levels in N22oe-33, 34, and 37 lines were 27, 22, and 11 times higher than that in WT plants, respectively (**Figure 4C**). During the experiments under normal watered condition in greenhouse, we noticed that the N22oe plants showed growth retardation (**Figure 4D**), leading to 25–29% of reduction in plant height (**Figure 4E**), as compared to WT plants. In addition, we also noted that the panicles of the N22oe plants grown under greenhouse condition were smaller than the WT plants (**Figure 4F**), leading to significant reductions in grain numbers per panicle and 1000-grain weight (**Figures 4G,H**). These data indicate that overexpression of *ONAC022* has a negative effect on growth and a penalty on grain yield in transgenic rice. No significant difference in other morphological and developmental characters such as tiller number, flowering and heading was observed between the N22oe and WT plants.

### Increased Drought and Salt Tolerance in N22oe Plants

**FIGURE 5:**
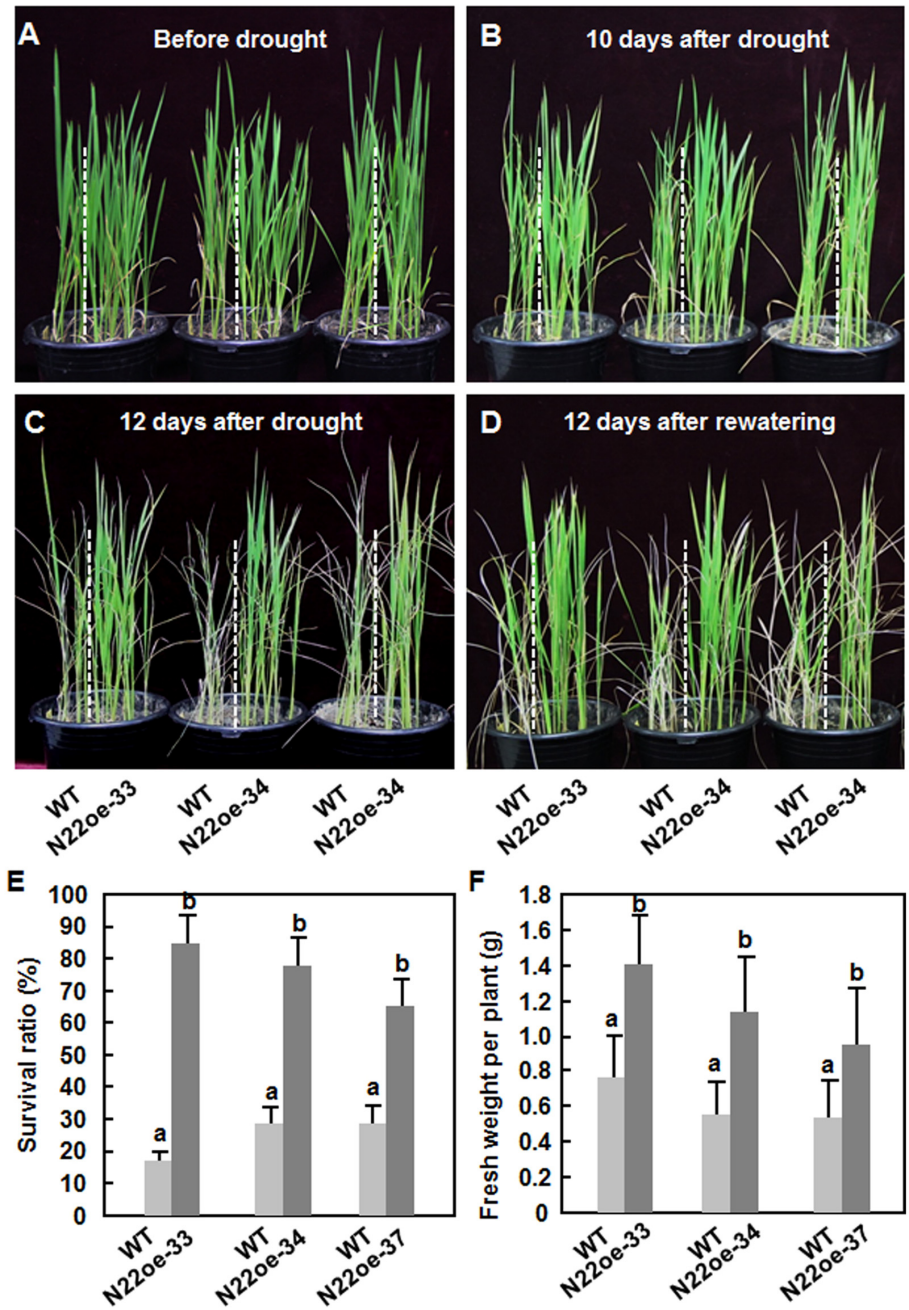
**Increased drought tolerance in N22oe plants. (A–D)** Phenotype of the N22oe and WT seedlings at different stages during the drought stress experiments. The N22oe seedlings were grown in barrels each with WT seedlings as a control. **(E)** Survival ratios of the N22oe and WT plants at 12 days after re-watering. **(F)** Growth biomass of the seedlings after drought stress treatment. Data presented in **(E,F)** are the mean ± SD from three independent experiments and different letters above the columns indicate significant differences at *p* ≤ 0.05 level.

To examine whether *ONAC022* plays a role in abiotic stress tolerance, we compared the drought and salt tolerance of the N22oe and WT plants at vegetative growth stage. Before drought stress treatment, the N22oe and WT seedlings exhibited similar growth status (**Figure 5A**). The WT seedlings started to show leaf rolling at 10 days (**Figure 5B**) and became severe leaf rolling and wilting at 12 days after withholding water (**Figure 5C**). During the drought stress process, however, the N22oe seedlings showed delayed and less leaf rolling and did not show wilting symptom during the drought stress process, as compared with the WT seedlings (**Figures 5B,C**). After re-watering regularly for 12 days, only 24% of the WT seedlings were recovered, while 85, 78, and 66 of the N22oe-33, 34, and 37, respectively, survived (**Figures 5D,E**). Similarly, the fresh weights of individual N22oe plant were significantly higher than WT at 12 days after recovery, giving approximately 50% of increase (**Figure 5F**). These data demonstrate that the N22oe plants exhibit increased tolerance to drought stress and that *ONAC022* plays a critical role in rice drought stress tolerance.

**FIGURE 6:**
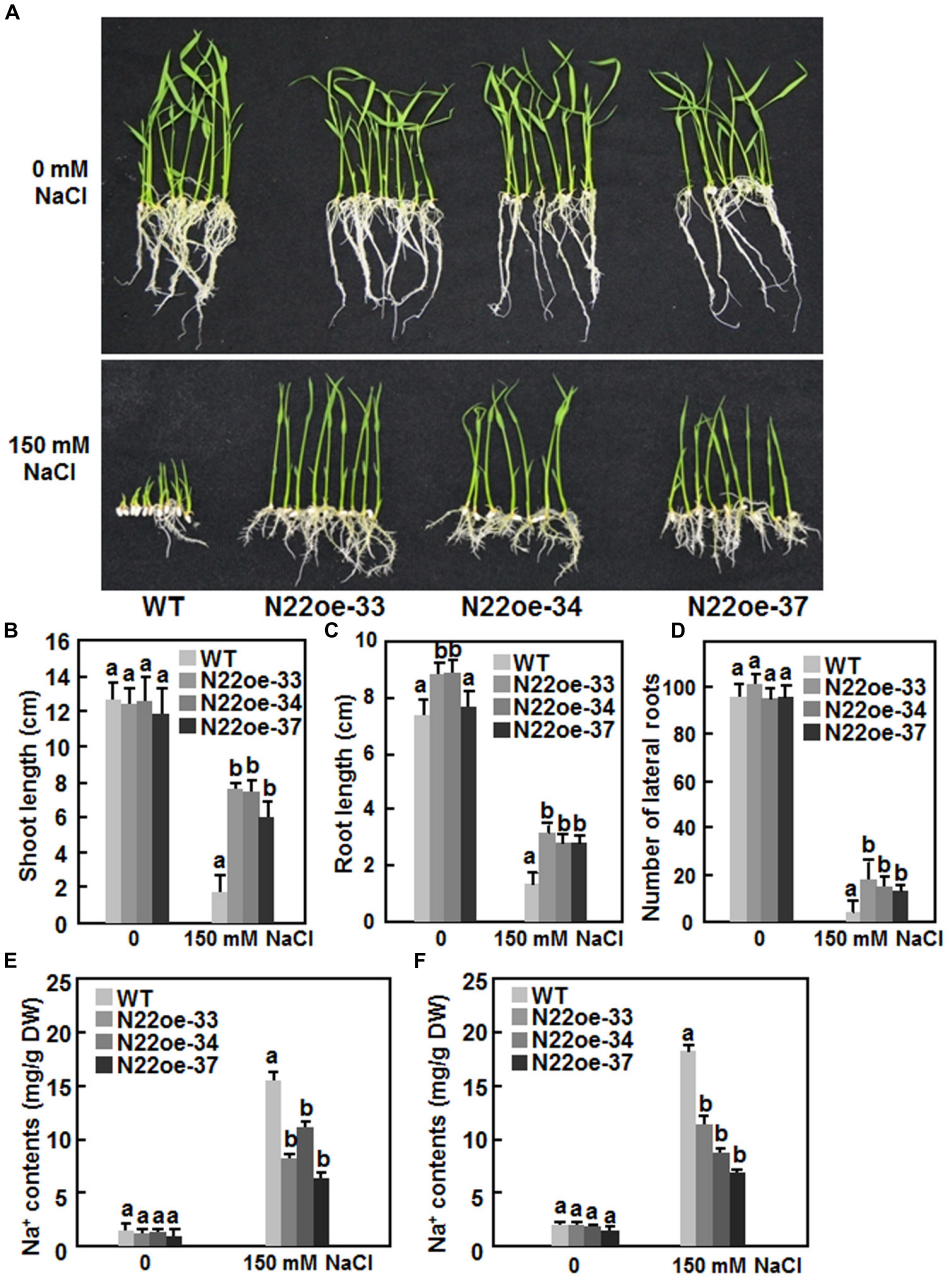
**Increased salt tolerance in N22oe plants. (A)** Growth performance of the N22oe and WT seedlings grown on 1/2 MS medium supplemented with or without 150 mM NaCl. **(B,C)** Shoot and root length of the N22oe and WT seedlings grown on 1/2 MS medium supplemented with or without 150 mM NaCl. **(D)** Number of lateral roots of the N22oe and WT seedlings grown on 1/2 MS medium supplemented with or without 150 mM NaCl. **(E,F)** Na^+^ contents in roots and shoots of the N22oe and WT seedlings grown in modified Hogland solution with or without 150 mM NaCl. Data presented in **(B–F)** are the mean ± SD from three independent experiments and different letters above the columns indicate significant differences at *p* ≤ 0.05 level with corresponding WT.

We next compared the salt tolerance of the N22oe and WT plants. No significant difference between the N22oe and WT lines was observed for seed germination and seedling growth on 1/2 MS medium without supplement of NaCl (**Figure 6A**, upper). However, in the presence of 150 mM NaCl, seed germination and seedling growth of the WT were markedly inhibited at 7 days after planting on 1/2 MS medium. By contrast, seeds of the N22oe-33, 34, and 37 lines germinated and the seedlings grew normally on 1/2 MS medium supplemented with 150 mM NaCl (**Figure 6A**, lower) although the seedling growth of the N22oe lines was inhibited to some extent as compared with those grown on NaCl-free medium (**Figure 6A**). The shoot length of the N22oe seedlings was similar to that of the WT seedlings when grown under normal condition, but was significantly higher than the WT seedlings when grown under NaCl stress, leading to twofold to threefold of increases (**Figure 6B**). The root length of the N22oe seedlings was also higher than that of the WT seedlings when grown under normal condition or NaCl stress and showed 70–85% increase over the WT seedlings when grown under NaCl stress condition (**Figure 6C**). Also, the lateral root numbers of the N22oe seedlings were much more than that of the WT seedlings when grown under NaCl stress condition, leading to 1.5- to 3-fold of increases (**Figure 6D**). We also measured the Na^+^ content in shoots and roots of WT and N22oe seedlings grown under normal or salt stress condition. When grown in normal Hogland solution, the Na^+^ content in roots and shoots showed no significant difference between WT and N22oe seedlings (**Figures 6E,F**). The Na^+^ contents in roots and shoots of plants grown in Hogland solution supplemented with 150 mM NaCl were significantly increased as compared to those in plants grown normal Hogland solution (**Figure 6E**). However, the Na^+^ content in roots and shoots of the N22oe plants were significantly reduced by 25–55 and 32–47, respectively, as compared to those in WT plants (**Figures 6E,F**). These results suggest that the N22oe plants show increased salt tolerance and that *ONAC022* functions importantly in rice salt tolerance.

### Increased Contents of Stress-Related Metabolites and Reduced Transpiration in N22oe Plants

**FIGURE 7:**
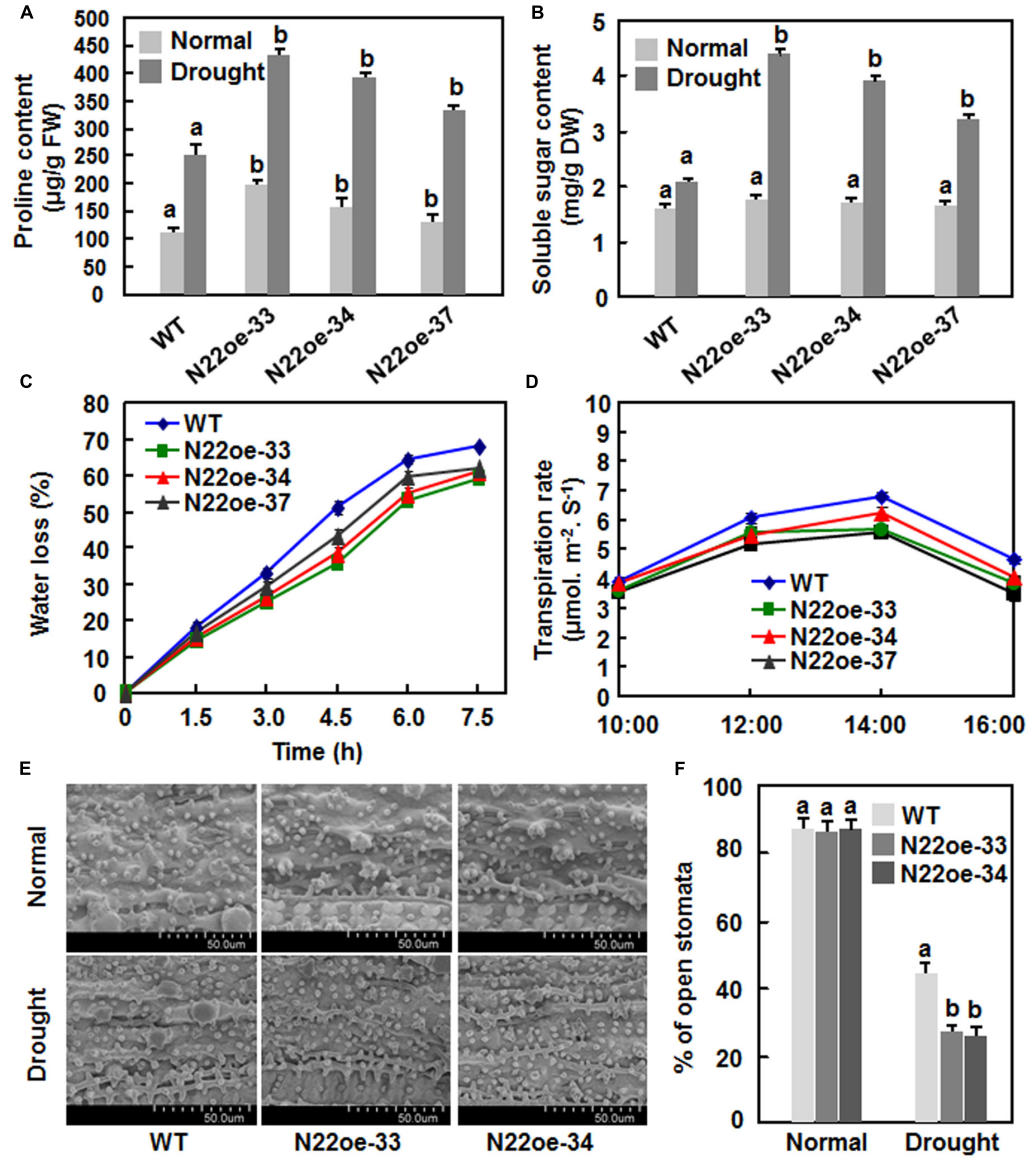
**Physiological changes inN22oe plants. (A)** Proline contents in leaves of the N22oe and WT plants grown under normal and drought stress condition. **(B)** Soluble sugar contents in leaves of the N22oe and WT plants grown under normal and drought stress condition. Four-week-old plants were subjected for drought stress treatment by stopping watering and leaf samples were collected at 6 days after drought treatment. **(C)** Rates of water loss in detached leaves of the N22oe and WT plants. Leaves were detached from 4-week-old N22oe and WT plants grown under normal conditions and placed on lab bench for drought treatment. Leaf samples were collected at indicated time points and subjected for measuring water loss. **(D)** Transpiration rate of the N22oe and WT plants. Transpiration rates in six flag leaves of 3-month-old N22oe and WT plants grown under greenhouse condition were determined by Li-6400 instrument at indicated time points. **(E)** Stomatal aperture of the N22oe and WT plants grown under normal and drought condition. Scale bar = 50 μM. **(F)** Percentage of open stomata in leaves of the N22oe and WT plants grown under normal and drought condition. Data presented in **(A–D,F)** are the mean ± SD from three independent experiments and different letters above the columns indicate significant differences at *p* ≤ 0.05 level with corresponding WT.

To explore the possible physiological mechanism responsible for the increased drought and salt tolerance in N22oe plants, we compared some stress-related physiological changes between the N22oe and WT plants grown under normal and drought conditions. Under normal growth condition, the proline contents in the N22oe plants were markedly higher than the WT plants, leading to 20–78% of increases (**Figure 7A**), whereas no significant difference in the contents of soluble sugars was observed between the N22oe and WT plants (**Figure 7B**). At 10 days after drought stress treatment, the proline and soluble sugar contents were increased significantly both in N22oe and WT plants as compared with those in plants grown under normal condition (**Figures 7A,B**). However, the increases in proline and soluble sugar contents in N22oe plants were much evident than those in WT plants under drought stress condition, resulting in 32–73 and 55–110% of increases for the proline and soluble sugar contents, respectively (**Figures 7A,B**). The rates of water loss in detached leaves of the N22oe plants were approximately 8–15% lower than those in detached leaves of the WT plants during a period of 3–7.5 h after detachment (**Figure 7C**). Similarly, the transpiration rates in leaves of the N22oe plants under normal sunlight condition were reduced by 0.5–1.17%, as compared with those in WT plants, at 12:00, 14:00, and 16:00 (**Figure 7D**). Furthermore, we also examined the stomatal density and behavior between the N22oe and WT plants grown under normal and drought stress conditions. No difference in stomatal density was observed (**Figure 7E**) between the N22oe and WT plants, but the percentages of open stomata in leaves of the N22oe plants grown under drought condition were significantly lower than that in the WT plants (**Figure 7F**). These results indicate that increased contents of stress-related metabolites, reduced transpiration rates and less open stomata may be the causes that confer the increased drought tolerance in the N22oe plants.

### Increased ABA Sensitivity in N22oe Plants

**FIGURE 8:**
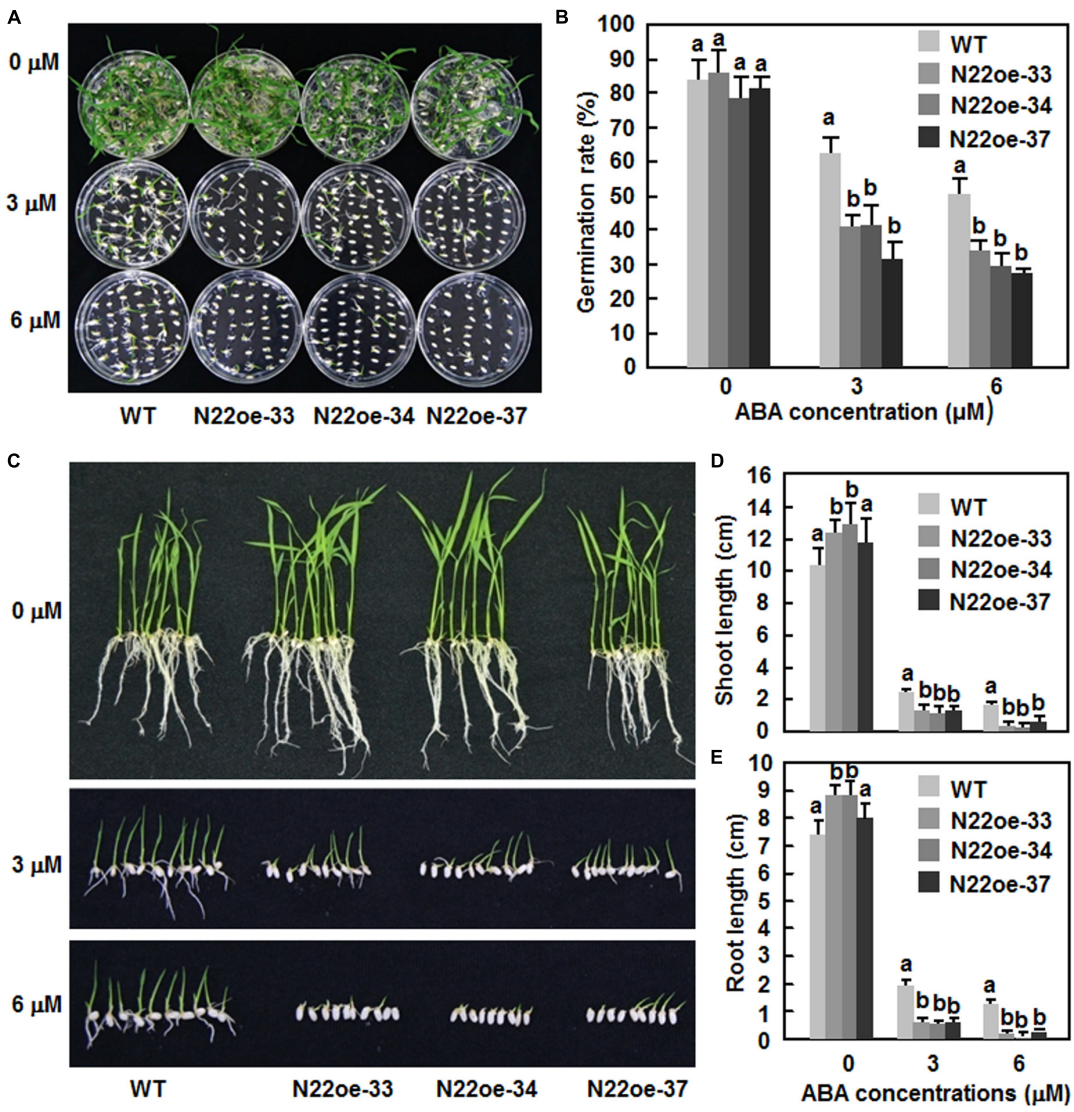
**Increased ABA sensitivity of the N22oe seedlings. (A,C)** Growth performance and **(B)** germination rate of the N22oe and WT seedlings on 1/2 MS medium supplemented with or without different concentrations of ABA. Photos were taken at 6 days after germination. **(D,E)** Shoot and root lengths of the N22oe and WT seedlings grown on 1/2 MS medium supplemented with or without different concentrations of ABA. The shoot and root lengths were measured at 6 days after germination. Data presented in **(D,E)** are the mean ± SD from three independent experiments and different letters above the columns indicate significant differences at *p* ≤ 0.05 level with corresponding WT.

The ABA sensitivity of the N22oe lines was examined and compared with WT by analyses of seed germination and seedling growth. On 1/2 MS medium without ABA, seeds of the N22oe and WT lines germinated normally and no significant difference was observed between the N22oe and WT lines (**Figures 8A,B**). However, on 1/2 MS medium supplemented with 3 μM or 6 μM ABA, germination of seeds of the N22oe lines was significantly inhibited as compared with the WT line (**Figures 8A,B**), resulting in 35–54% of reduction in seed germination rate as compared with the WT at the same ABA concentration (**Figure 8B**). Similarly, the N22oe seedlings grew better than those of the WT seedlings on 1/2 MS medium without ABA (**Figure 8C**) and had longer shoots and roots, leading to 17–27 and 11–23% of increases for shoot and root lengths (**Figures 8D,E**). However, significant growth inhibition of the N22oe seedlings grown on 1/2 MS medium with 3 μM or 6 μM ABA was observed (**Figure 8C**), resulting in 45–52 and 67–85% of reduction for shoot length at 3 μM or 6 μM ABA and 66–72 and 83–93% of decrease for root length at 3 μM or 6 μM ABA, respectively, as compared with the WT seedlings (**Figures 8D,E**). These data indicate that overexpression of *ONAC022* results in increased ABA sensitivity of the transgenic rice.

### Increased ABA Biosynthesis and Contents in N22oe Plants

**FIGURE 9:**
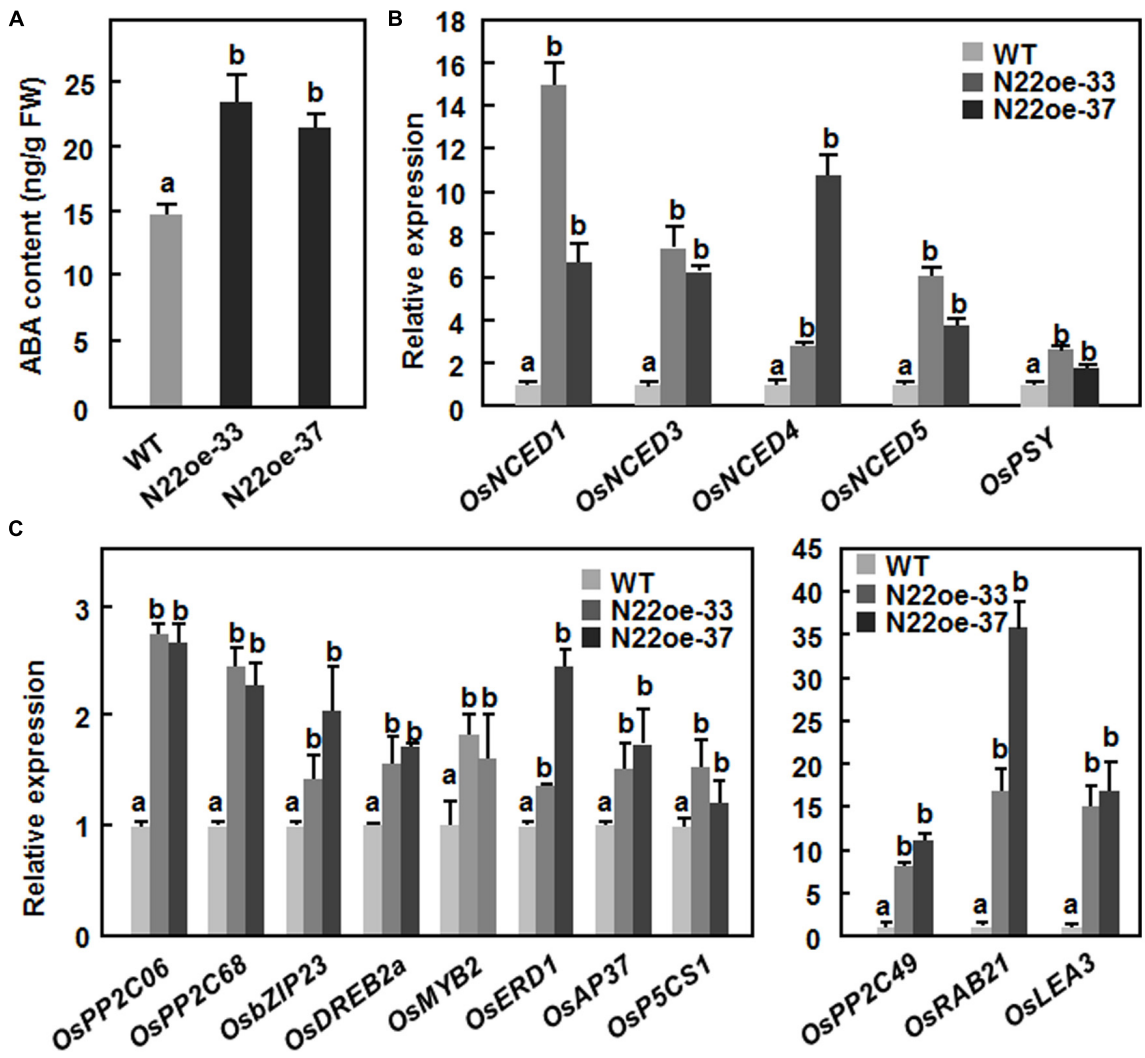
**Increased ABA content and upregulated expression of ABA biosynthetic and stress-responsive genes in N22oe plants. (A)** Increased ABA contents in the N22oe plants. **(B)** Expression of ABA biosynthesis-related genes in the N22oe plants. **(C)** Expression of stress-responsive genes in the N22oe plants. Leaf samples were collected from 30-day-old seedlings grown under normal condition and subjected for analyses of ABA content and gene expression. ABA in leaf samples was extracted and quantified by HPLC. FW, fresh weight. Expression of the ABA biosynthetic and stress-responsive genes was analyzed by qRT-PCR and relative expression levels were shown as folds of the level of the actin gene. Data presented are the mean ± SD from three independent experiments and different letters above the columns indicate significant differences at *p* ≤ 0.05 level with corresponding WT.

The increased ABA sensitivity in the N22oe plants led us to examine whether overexpression of *ONAC022* affects the endogenous level of ABA and its biosynthesis in transgenic rice. As shown in **Figure 9A**, the endogenous ABA content in the N22oe-33 and 37 plants was significantly higher than that in the WT plants, leading to 60 and 46% of increases, respectively. Meanwhile, the expression of several genes involved in ABA biosynthesis such as *OsNCED1*, *OsNCED3*, *OsNCED4*, *OsNCED5*, and *OsPSY* was upregulated in the N22oe plants grown under normal condition, resulting in increases of 2.8- to 14.9-fold for *OsNCEDs* and 1.7- to 2.6-fold for *OsPSY* over those in WT plants (**Figure 9B**). These results suggest that overexpression of *ONAC022* can upregulate the expression of many ABA biosynthesis-related genes and thus increase endogenous ABA content in the N22oe plants.

### Upregulated Expression of ABA- and Stress-Responsive Genes in N22oe Plants

To gain further insights into the mechanism of the increased drought and salt tolerance in N22oe plants, the gene expression profiles between 3-week-old N22oe and WT plants grown under normal condition were determined and compared using the Affymetrix rice gene chip. A total of 1059 genes were identified as differentially expressed genes (486 up-regulated and 573 down-regulated) that showed a >twofold change in the transcript levels in N22oe plants (Supplementary Figure S1), compared with the transcript levels in WT plants, including a number of stress-responsive genes encoding for protein phosphatase 2Cs (LOC_Os11g01790 and LOC_Os12g01770), late embryogenesis abundant proteins (LOC_Os03g20680 and LOC_Os01g50910) and bZIP protein (LOC_Os02g14910; Supplementary Table S2). Furthermore, we selected 11 stress-responsive genes and compared their expression in the N22oe and WT plants grown under normal conditions. As shown in **Figure 9C**, the expression levels of *OsPP2C06*/*OsABI2*, *OsPP2C49*, and *OsPP2C68*, three members of the PP2C family known to be involved in abiotic stress tolerance (Singh et al., 2010), were up-regulated in the N22oe plants, showing increases of 1.3- to 10.5-fold over those in WT plants. Several stress-related TF genes such as *OsbZIP23* (Xiang et al., 2008), *OsDREB2a* (Dubouzet et al., 2003), *OsMYB2* (Yang et al., 2012), and *OsAP37* (Oh et al., 2009) were up-regulated in the N22oe plants, giving increases of 0.34- to 1.51-fold over those in WT plants (**Figure 9C**). Furthermore, the expression levels of some stress-related genes like *OsRAB21* (Xiong et al., 2014), *OsLEA3* (Xiao et al., 2007), *OsERD1* (a homolog of *Arabidopsis AtERD1*; Kiyosue et al., 1994), and *OsP5CS1* (Xiong et al., 2014) in N22oe plants were also upregulated with increases of 0.3- to 1.4-fold for *OsERD1* and *OsP5CS1* and 14- to 35-fold for *OsRAB21* and *OsLEA3* over those in WT plants (**Figure 9C**). These data indicate that overexpression of *ONAC022* in N22oe plants, affects the expression of a set of stress-related genes and thus confers an increased drought and salt tolerance in transgenic rice.

## Discussion

The NAC TFs represent a quite large family with 151 members in rice (Fang et al., 2008; Nuruzzaman et al., 2010). Although at least 10 rice NAC genes such as *ONAC002* (*SNAC1*/*OsNAC9*; Hu et al., 2006; Redillas et al., 2012), *ONAC068* (*OsNAC4*; Kaneda et al., 2009), *ONAC048* (*SNAC2*/*OsNAC6*; Nakashima et al., 2007; Hu et al., 2008), *ONAC009* (*OsNAC5*; Takasaki et al., 2010; Song et al., 2011; Jeong et al., 2013), *ONAC058* (*OsNAP*; Zhou et al., 2013; Chen et al., 2014; Liang et al., 2014), *ONAC122* (*OsNAC10*; Jeong et al., 2010; Sun et al., 2013), *ONAC131* (Sun et al., 2013), *ONAC054* (*RIM1*; Yoshii et al., 2009), *ONAC045* (Zheng et al., 2009), and *ONAC017* (*OsNAC111*; Yokotani et al., 2014) have been shown to play important roles in abiotic and biotic stress responses; however, the biological function of most *ONAC* genes is not known. In the preset study, we demonstrated that ONAC022 is a stress-responsive NAC with transcriptional activator activity and play an important role in drought and salt stress tolerance in rice. The *Arabidopsis* ANAC036, the closest ortholog of ONAC022, was found to be involved in the growth of leaf cells (Kato et al., 2010) but its role in abiotic stress response remains unclear.

Our previous comprehensive analysis using publicly available microarray expression data identified a total of 63 *ONAC* genes, including *ONAC022*, that exhibited overlapping expression patterns in rice under various abiotic (e.g., salt and drought) and biotic (e.g., infection by fungal, bacterial, and viral pathogens) stresses (Sun et al., 2015). In this study, we further verified that the expression of *ONAC022* was induced significantly by drought, salt, and ABA (**Figure 2**), indicating that *ONAC022* can respond to multiple environmental cues. This is partially supported by the fact that the promoter region of the *ONAC022* gene contains several stress-related *cis*-elements such as ABREs and GCC box (**Figure 1C**), which are present in promoters of stress-responsive genes that are regulated by DREB and ERF TFs, respectively (Yamaguchi-Shinozaki and Shinozaki, 2005, 2006; Mizoi et al., 2012; Licausi et al., 2013). The ONAC022 protein can bind specifically to a canonical NAC recognition *cis*-element fragment wNACRS *in vitro* (Xie et al., 2000; Trans et al., 2004) and has transcriptional activity that is dependent on its C-terminal (**Figures 3B,C**). This is in agreement with a common knowledge that NAC proteins have a C-terminal transcription activation domain (Hu et al., 2006; Takasaki et al., 2010; Liang et al., 2014). The C-terminal region of ONAC022 contains a newly identified C1 domain (**Figure 1A**), which comprises the putative NAC subdomain E and its immediately downstream sequence, indicating that the C1 domain in ONAC022 may be involved in the DNA-binding ability (Duval et al., 2002; Kato et al., 2010).

The observations that the N22oe plants showed improved drought and salt stress tolerance as revealed by higher survival ratio and better growth performance under drought and salt stress condition (**Figures 5** and **6**) demonstrate that ONAC022 is a positive regulator of drought and salt stress tolerance in rice. Several other rice NAC genes including *ONAC001* (*SANC1*/*OsNAC9*; Hu et al., 2006; Redillas et al., 2012), *ONAC048* (*SNAC2*/*OsNAC6*; Hu et al., 2008; Takasaki et al., 2010; Song et al., 2011; Jeong et al., 2013), *ONAC009* (*OsNAC6*; Nakashima et al., 2007), *ONAC122* (*OsNAC10*; Jeong et al., 2010), *ONAC058* (*OsNAP*; Chen et al., 2014), and *ONAC045* (Zheng et al., 2009) were previously reported to play positive roles in improving drought and salt tolerance when overexpressed in transgenic rice. The mechanisms responsible for the improved drought and salt tolerance in the N22oe plants can be, at least partially, explained by several morphological, physiological, and biochemical changes observed in the present study. Firstly, accumulation of compatible solutes such as soluble sugars and free proline, which act as osmolytes to facilitate osmo-regulation, molecular chaperones to stabilize proteins or regulators of the antioxidant system, is a common phenomenon in response to abiotic stress (Liu and Zhu, 1997; Garg et al., 2002). It was observed that the N22oe plants accumulated higher levels of free proline under normal condition and high levels of free proline and soluble sugars under drought stress condition, than the WT plants (**Figures 7A,B**). High levels of free proline in the N22oe plants may due to an upregulated expression of *OsP5CS1* (**Figure 9C**), whose overexpression in transgenic rice led to stress-induced accumulation of free proline and increased abiotic stress tolerance (Zhu et al., 1998). Thus, increased accumulation of free proline and soluble sugars in the N22oe plants may partially account for the improved drought and salt tolerance. In this context, similar observations have also been observed in transgenic rice overexpressing *ONAC009* (*OsNAC5*) and *ONAC058* (*OsNAP*; Song et al., 2011; Chen et al., 2014). Secondly, reduced rates of water loss in detached leaves, decreased transpiration rate and increased stomatal closure in leaves of whole plants were observed in the N22oe plants (**Figures 7C,D**), which may be the factors that contribute to the improved drought and salt tolerance in the N22oe plants. It is well known that stomatal behavior and/or stomatal density on leaves of plants affect greatly the rates of water loss and transpiration under drought stress condition. For example, it was previously found that increased stomatal closure at the early stage of drought stress was an important factor causing reduced rates of transpiration/water loss and improved drought tolerance in the *ONAC002* (*SNAC1*)-overexpressing rice (Hu et al., 2006). *SNAC1* was induced strongly in guard cells of rice plants, suggesting that increased stomatal closure is likely a target of regulation by *ONAC002* (*SNAC1*; Hu et al., 2006). However, whether *ONAC022* plays a role in regulating stomatal behavior under drought stress condition and density and thereby contributes to the increased drought tolerance of the N22oe plants need to be examined further. Thirdly, it is well accepted that root system size correlates with the tolerance to water stress and that a longer root system should facilitate water absorption from soils and thus strengthen drought tolerance under water-deficit conditions. For example, drought-resistant rice varieties have a larger and more highly branched root system than drought-sensitive varieties (Price et al., 1997). We also noted that the N22oe plants had larger root system, as revealed by longer roots and more lateral roots than the WT plants, when grown under salt stress condition (**Figure 6**), indicating a possible role for *ONAC022* in regulating root system under stress condition and thereby improving stress tolerance. This is consistent with several observations that overexpression of *ONAC122* (*OsNAC10*; Jeong et al., 2010), *ONAC002* (*OsNAC9*; Redillas et al., 2012), and *ONAC009* (*OsNAC5*; Jeong et al., 2013) in transgenic rice and *ONAC002* (*SNAC1*; Liu et al., 2014) in transgenic cotton resulted in enhanced root system or altered root architecture involving an enlarged stele and aerenchyma but is contrary to the observation that the *ONAC002* (*SNAC1*)-overexpressing rice plants had no difference from WT plants in terms of root depth and volume (Hu et al., 2006).

Abscisic acid plays a critical role in regulating abiotic stress response in plants. In the present study, our results suggest that *ONAC022* may function as a positive regulator of stress response through modulating an ABA-mediated pathway to improve drought and salt tolerance in the N22oe rice. This hypothesis is supported by several lines of evidence presented in this study. Firstly, expression of *ONAC022* was significantly induced by exogenous ABA (**Figure 2C**), similar to several previously reported stress-responsive rice NAC genes such as *ONAC122* (*OsNAC10*; Jeong et al., 2010), *ONAC048* (*SNAC2*/*OsNAC6*; Nakashima et al., 2007; Hu et al., 2008), *ONAC009* (*OsNAC5*; Takasaki et al., 2010; Song et al., 2011), *ONAC058* (*OsNAP*; Chen et al., 2014; Liang et al., 2014), and *ONAC002* (*SNAC1*; Hu et al., 2006). Secondly, the N22oe plants exhibited an increased ABA sensitivity than the WT in terms of seed germination and seedling growth (**Figure 8**). This is consistent with the observations that overexpression of *ONAC058* (*OsNAP*; Chen et al., 2014), *ONAC002* (*SNAC1*; Hu et al., 2006), and *ONAC048* (*SNAC2*; Hu et al., 2008) in transgenic rice led to improved abiotic stress tolerance and hypersensitivity to exogenous ABA. Thirdly, the endogenous ABA content in the N22oe plants was higher than that in WT plants (**Figure 9A**), accompanying with upregulated expression of some of the ABA biosynthesis-related genes such as *OsNCEDs* (**Figure 9B**), which is generally considered to be the rate-limiting step in the stress-induced ABA biosynthesis pathway (Qin and Zeevaart, 1999; Taylor et al., 2000; Iuchi et al., 2001). These data indicate that overexpression of *ONAC022* may influence the biosynthesis of ABA via modulating directly or indirectly the expression of ABA biosynthesis-related genes. This is similar to the observations that the root-specific overexpression of *ONAC002* (*OsNAC9*) and *ONAC122* (*OsNAC10*) significantly upregulated the expression level of *OsNCEDs* in transgenic rice (Jeong et al., 2010; Redillas et al., 2012) and that overexpression of *OsMYB481*, a stress-responsive MYB TF, led to a significant increase in expression of ABA biosynthetic genes and a high level of the endogenous ABA in transgenic rice plants (Xiong et al., 2014). Generally, as a critical stress hormone, high level of the endogenous ABA might strengthen and/or accelerate stress response and thus correlates with improved abiotic stress tolerance. For example, mutation in *OsDSM2*/*OsBCH1*, one of three putative β-carotene hydroxylases that are predicted for the biosynthesis of ABA precursor zeaxanthin, resulted in reduced level of the endogenous ABA and decreased drought stress tolerance (Du et al., 2010), whereas RNAi-mediated suppression of *OsABA8ox3*, one of three ABA 8′-hydroxylases involved in catabolism of ABA, led to a high level of the endogenous ABA and improved drought stress tolerance (Cai et al., 2015). Lastly, the increased endogenous ABA levels in plants will generally initiate ABA-mediated pathway that regulates the expression of many stress-responsive genes (Xiong et al., 2002). In the present study, we observed that a large number of the stress-responsive genes were differentially expressed in N22oe plants (**Figure 9C**) and the expression of some early ABA signaling and regulatory genes such as *OsPP2C06*/*49*/*68* (Singh et al., 2010), *OsbZIP23* (Xiang et al., 2008), *OsDREB2a* (Dubouzet et al., 2003), *OsMYB2* (Yang et al., 2012), and *OsAP37* (Oh et al., 2009) and late ABA-responsive stress-related genes such as *OsRAB21* (Xiong et al., 2014), *OsLEA3* (Xiao et al., 2007; Duan and Cai, 2012), and *OsP5CS1* (Xiong et al., 2014) was significantly upregulated in the N22oe plants (**Figure 9C**), demonstrating an activated ABA-mediated pathway in the transgenic plants. Overall, these data suggest that *ONAC022* in the N22oe plants may accelerate the ABA synthesis via modulating the expression of the ABA biosynthesis-related genes and thus activate an ABA-mediated pathway to regulate the expression of stress-responsive genes. However, it cannot be ruled out the possibility that *ONAC022* regulates abiotic stress response through an ABA-independent pathway because the expression of *OsERD1*, which is known to be involved in the ABA-independent pathway (Shinozaki and Yamaguchi-Shinozaki, 2007), was also upregulated significantly in the N22oe plants (**Figure 9C**).

Growth retardation has been observed in transgenic rice plants overexpressing stress-responsive TFs such as *ONAC048* (*OsNAC6*) in rice (Nakashima et al., 2007). In the present study, we also noticed that the N22oe plants showed a stunted growth phenotype and smaller panicles compared with the WT plants (**Figure 4**), indicating that overexpression of *ONAC022* in transgenic rice has adverse effects on growth and yield formation. This is similar to the observation that overexpression of *ANAC036*, the closest ortholog of rice *ONAC022*, in *Arabidopsis* led to a dwarf phenotype by reducing cell size in leaves and stems (Kato et al., 2010) but differs from several reports that overexpression of *ONAC058* (*OsNAP*), *ONAC002* (*OsNAC9*), and *ONAC122* (*OsNAC10*) in transgenic rice had no significant effects on growth under normal condition and improved yield production under drought stress condition (Jeong et al., 2010; Redillas et al., 2012; Chen et al., 2014). The fact that a large portion of genes encoding proteins involved in developmental and reproduction processes were differentially expressed in N22oe plants (Supplementary Figure S1) supports the involvement of *ONAC022* in rice growth and development. However, the adverse effects of *ONAC022* on rice growth and panicle development may be due to re-allocation of energy between stress tolerance and normal growth/development, and the mechanisms need to be further investigated.

In summary, results presented in this study demonstrate that ONAC022 functions as a stress-responsive transcriptional activator and overexpression of *ONAC022* in transgenic rice can significantly improve drought and salt stress tolerance through an ABA-mediated pathway. Our findings presented in this study, together with several previous reports (Hu et al., 2006, 2008; Jeong et al., 2010; Chen et al., 2014), demonstrate that overexpression of a single regulatory gene such as *ONAC022* is a promising strategy to improve the abiotic stress tolerance in rice and other commercially important crops. However, functional analysis with knockout mutants is necessary to determine whether *ONAC022* is required for abiotic stress tolerance in rice. Further investigations on the identification of *ONAC022*-regulated target genes will be helpful to elucidate the mechanism of *ONAC022* in regulating abiotic stress tolerance. As the N22oe plants gain an improved drought and salt tolerance with a penalty on grain production under unstressed condition, it is necessary to evaluate in detail their performance on abiotic stress tolerance and grain yield under natural or stressed conditions before consideration of their potential use as novel materials in breeding program.

## Author Contributions

YH, HZ, DL, and FS designed the research; YH, HZ, LH, and DL performed the experiments; YH and FS wrote the manuscript.

## Conflict of Interest Statement

The authors declare that the research was conducted in the absence of any commercial or financial relationships that could be construed as a potential conflict of interest.
