# Supplementary material for: Overexpression of a Stress-Responsive NAC Transcription Factor Gene ONAC022 Improves Drought and Salt Tolerance in Rice
Source: Front Plant Sci. 2016 Jan 22;7:4. doi: 10.3389/fpls.2016.00004 (PMC4722120; doi:10.3389/fpls.2016.00004)
Supplement: Supplementary file 1 [file Table_1.DOC]

**Table S1. Primers and oligos used in this study**

| Name | Primer sequence (5’-3’) |
| --- | --- |
| ***Plasmid constrcutions*** | |
| ONAC022-F-OE | ATGGCGATGACACCGCAGCT |
| ONAC022-R-OE | GAG AAGCTT TTAGTAGTAGAGCAGATGGG |
| ONAC022-R-srdx | GAG AAGCTT GTAGTAGAGCAGATGGGCGT |
| ONAC022-F (GFP) | AGT GGA TCC ATGGCGGCGGCAGTGGC |
| ONAC022-R (GFP) | GCG TCT AGA TTAGTAGTAGAGCAGATGGGCG |
| ONAC022-F (pBD) | AGT GAA TTC ATGGCGGCGGCAGTGGC |
| ONAC022ΔC-R (pBD) | AGT GTC GAC GGCGCCGTAGTCGGGGAGGC |
| ONAC022ΔN-R (pBD) | AGT GAA TTC CGCCCGCGCCGCCGCGC |
| ONAC022-R (pBD) | AGT GTC GAC TTAGTAGTAGAGCAGATGGGCG |
| ONAC022-GEX-F | GGAGGATCCATGGCGGCGGCAGTGGCTGT |
| ONAC022-GEX-R | CCGGAATTCGTAGTAGAGCAGATGGGCGT |
| ***Electrophoretic mobility shift assay*** | |
| NACRS-BIO-1 | ATCGCATGTGGAGCACGGAGCACGTTTT |
| NACRS-BIO-2 | AAAACGTGCTCCGTGCTCCACATGCGAT |
| mNACRS-BIO-1 | ATCGAAAAAAGAGAAAAGAGAAAATTTT |
| mNACRS-BIO-2 | AAAATTTTCTCTTTTCTCTTTTTTCGAT |
| ***Southern blotting*** | |
| HptII-Probe-F | ACACAGCCATCGGTCCAGAC |
| HptII-Probe-R | ATCTTAGCCAGACGAGCGGG |
| ***qRT-PCR*** | |
| ONAC022-RT-F | AAGGAGGACATGGTGCTCTGCAAG |
| ONAC022-RT-R | TGCATCTCTTCCATTGCCGAGGCT |
| OsActin-RT-F | AGCTGCGGGTATCCATGAGA |
| OsActin-RT-R | GCAATGCCAGGGAACATAGTG |
| OsNCED1-RT-F | TCCAGTCACAGCACCAATGATAC |
| OsNCED1-RT-F | TGTTCCACA GTAACTCGTAATTTCG |
| OsNCED3-RT-F | CCCCTCCCAAACCATCCAAACCGA |
| OsNCED3-RT-F | TGTGAGCATATCCTGGCGTCGTGA |
| OsNCED4-RT-F | GATTGCACGGCACCTTCATT |
| OsNCED4-RT-R | CTCTGTAATTTGATTTTTCACTGGCTAAT |
| OsNCED5-RT-F | CCCAGCTTGAAGCTTTTGCT |
| OsNCED5-RT-R | ACAACACTGCAACTATCCCTATCACT |
| OsPSY-RT-F | AGGCCAACGATTACAACAACTTC |
| OsPSY-RT-R | TAAGCTTTAGGCAGAGCCACAAT |
| OsbZIP23-RT-F | GGAGCAGCAAAAGAATGAGG |
| OsbZIP23-RT-R | GGTCTTCAGCTTCACCATCC |
| OsPP2C49-RT-F | GGCTTATTCTCTTCCTCCTCTAT |
| OsPP2C49-RT-R | AGTAAATTCTTTGCGACGATGAT |
| OsPP2C06-RT-F | GATGGTCTCTGGGACGTCAT |
| OsPP2C06-RT-R | CTTCGACAAGCACTCAGCAG |
| OsPP2C68-RT-F | CGCAGCTCCGACAACATCT |
| OsPP2C68-RT-R | GCTGGGTGACACTCTCTCTACAAG |
| OsMYB2-RT-F | GAGCAGCGAGGAGGAGGT |
| OsMYB2-RT-R | TGTAGTTGACGAGCAGGAGGT |
| OsAP37-RT-F | TCCGATGTTTTGGTCCTCTG |
| OsAP37-RT-R | TCCACGGTTTAGTCCATCTCATC |
| OsLEA3-RT-F | TGAAGAGCACGGTGGTCGG |
| OsLEA3-RT-R | GGCAGAGGTGTCCTTGTTGG |
| OsERD1-RT-F | TCAAAGGGAAGACGAAGCATGG |
| OsERD1-RT-R | GGGACGGAATACAACCATCTCA |
| OsP5CS1-RT-F | GCTGACATGGATATGGCAAAAC |
| OsP5CS1-RT-R | GTAAGGTCTCCATTGCATTGCA |
| OsRAB21-RT-F | CACACCACAGCAAGAGCTAAGTG |
| OsRAB21-RT-R | TGGTGCTCCATCCTGCTTAAG |
| OsDREB2a-RT-F | GGCTGAGATCCGTGAACCAA |
| OsDREB2a-RT-R | GGACCATACATTGCCCTTGC |
