# Supplementary material for: Overexpression of a Stress-Responsive NAC Transcription Factor Gene ONAC022 Improves Drought and Salt Tolerance in Rice
Source: Front Plant Sci. 2016 Jan 22;7:4. doi: 10.3389/fpls.2016.00004 (PMC4722120; doi:10.3389/fpls.2016.00004)
Supplement: Supplementary file 2 [file Table_2.DOC]

**Table S2. Partial list of upregulated genes in N22oe plants.**

| **Locus ID** | **Description** | **Folds (N22oe/WT)** |
| --- | --- | --- |
| LOC_Os01g72370 | helix-loop-helix protein | 3.6856 |
| LOC_Os07g44090 | myb protein Hv33 | 2.0798 |
| LOC_Os12g03150 | myb protein | 3.9448 |
| LOC_Os08g37730 | helix-loop-helix protein | 2.4101 |
| LOC_Os03g26210 | helix-loop-helix protein | 2.5804 |
| LOC_Os04g31290 | helix-loop-helix protein | 3.3214 |
| **LOC_Os02g14910** | **bZIP protein** | **2.3633** |
| LOC_Os03g52860 | lipoxygenase | 2.4831 |
| LOC_Os02g20360 | tyrosine aminotransferase | 3.347 |
| LOC_Os03g43100 | Peroxidase | 2.0196 |
| LOC_Os03g19427 | nicotianamine synthase | 145.713 |
| LOC_Os10g12080 | cytochrome P450 | 8.6366 |
| LOC_Os10g08319 | cytochrome P450 | 4.2048 |
| LOC_Os02g58720 | peroxidase precursor | 3.0771 |
| LOC_Os03g19420 | nicotianamine synthase | 52.896 |
| LOC_Os03g20680 | LEA1 | 14.5372 |
| LOC_Os01g50910 | LEA | 2.7952 |
| LOC_Os05g11990 | TTL1, response to abiotic stimulus | 2.4305 |
| **LOC_Os11g01790** | **protein phosphatase 2c** | **2.0165** |
| **LOC_Os12g01770** | **protein phosphatase 2c** | **2.3923** |
| LOC_Os07g08140 | heat stress transcription factor | 2.0591 |
| LOC_Os10g38540 | glutathione S-transferase | 2.6217 |
| LOC_Os03g28330 | sucrose synthase | 2.9471 |
| LOC_Os07g15460 | metal transporter Nramp6 | 4.8207 |
| LOC_Os01g68050 | amino acid transporter protein | 2.9855 |
| LOC_Os01g66010 | amino acid transporter | 4.302 |
| LOC_Os11g05390 | transporter, major facilitator family | 13.325 |
| LOC_Os01g68050 | amino acid transporter | 2.6462 |
| LOC_Os11g05390 | transporter, major facilitator family | 12.0433 |
| LOC_Os06g21250 | glycine rich protein family protein | 3.0181 |
| LOC_Os11g04020 | major facilitator superfamily antiporter | 12.8795 |
| LOC_Os03g09880 | AIR12 (Auxin response protein) | 2.8019 |
| LOC_Os02g13520 | OsIAA7 | 2.4061 |
| LOC_Os10g09990 | cytokinin-O-glucosyltransferase 3 | 2.1481 |
