# Supplementary material for: Overexpression of a Stress-Responsive NAC Transcription Factor Gene ONAC022 Improves Drought and Salt Tolerance in Rice
Source: Front Plant Sci. 2016 Jan 22;7:4. doi: 10.3389/fpls.2016.00004 (PMC4722120; doi:10.3389/fpls.2016.00004)

FIGURE S1 | Distribution of the differentially expressed genes (>2-fod of change in N22oe plants) among the biological process categories.

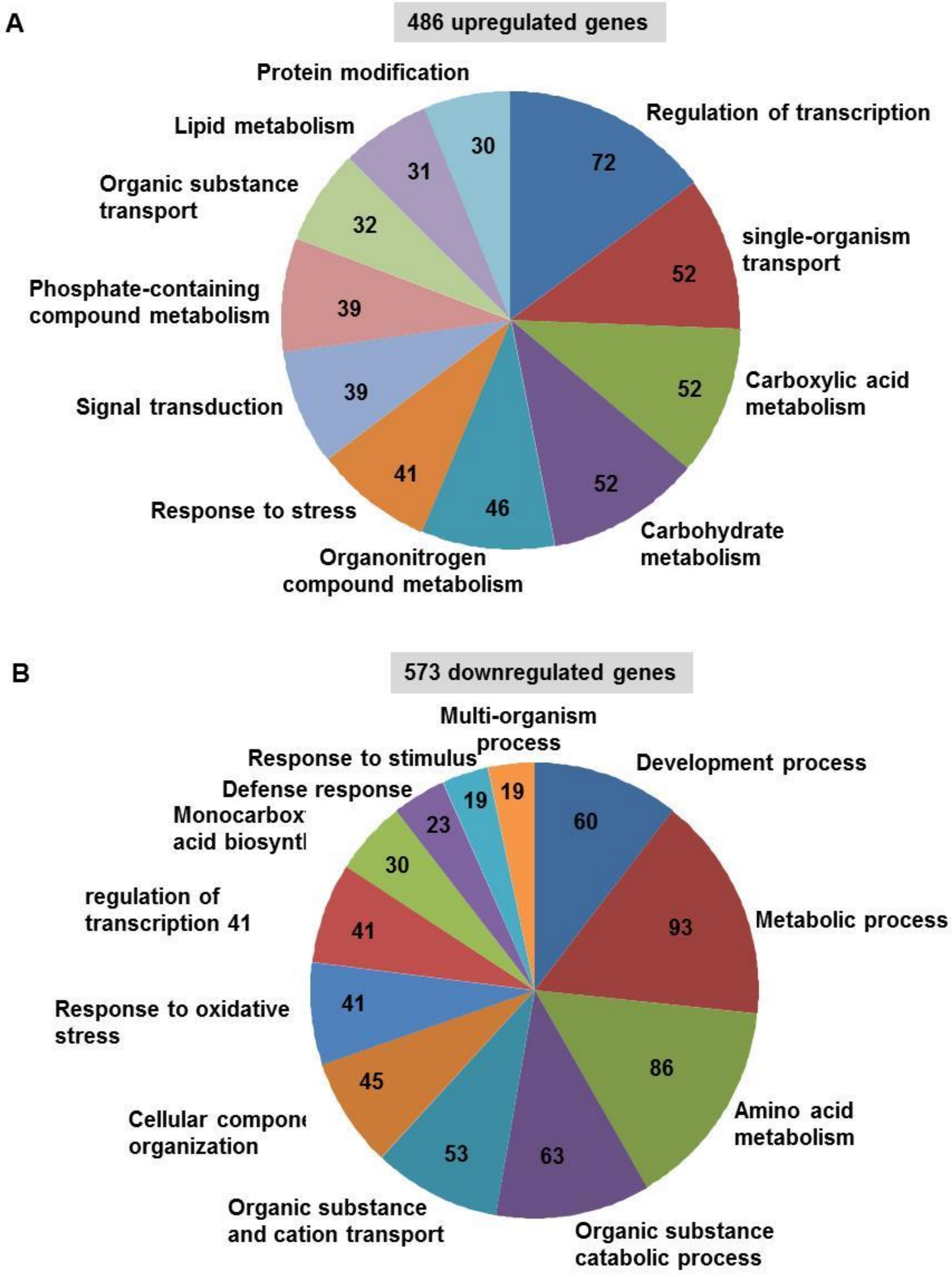

Supplement: Supplementary file 3 [file Presentation_1.PDF]
